# Supplementary material for: Identification and characterization of piperine synthase from black pepper, Piper nigrum L
Source: Commun Biol. 2021 Apr 8;4:445. doi: 10.1038/s42003-021-01967-9 (PMC8032705; doi:10.1038/s42003-021-01967-9)
Supplement: Supplementary file 2 — Supplementary Information [file 42003_2021_1967_MOESM2_ESM.pdf]

Supplementary Figures S1-S5

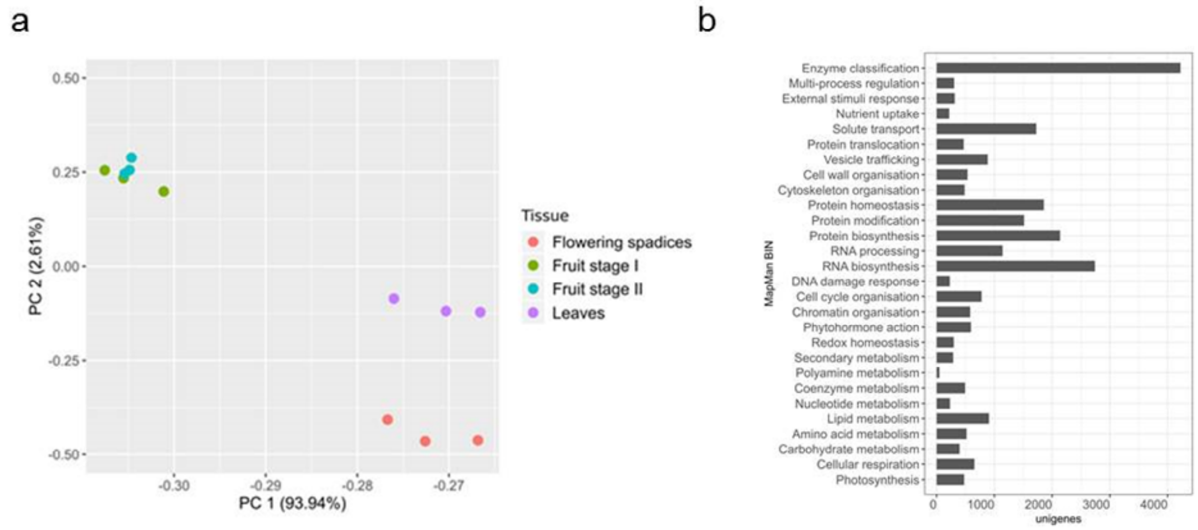

Supplementary Fig. S1: Principal component analysis and MapMan-based distribution[27] of transcripts. a Principal Component Analysis (PCA) was applied to 12 samples and 166,017 *Trinity* features using transformed gene expression data (counts per million). The first two principal components (PC) accounted for 96.56% of the total variance and identified three different clusters of leaf, spadices and fruit samples. PC1 accounted for 93.94% of the total variance and separates fruit sample from non-fruit samples. The high proportion of the captured variance in PC1 indicates large disparities in gene expression between fruits and all other black pepper tissues; **b** Distribution of functional categories of the transcript assembly comprising 208,307 transcript sequences. De-novo assembled sequences were annotated by Mercator/MapMan4 plant specific ontology. Unassigned (BIN 35) sequences were removed.

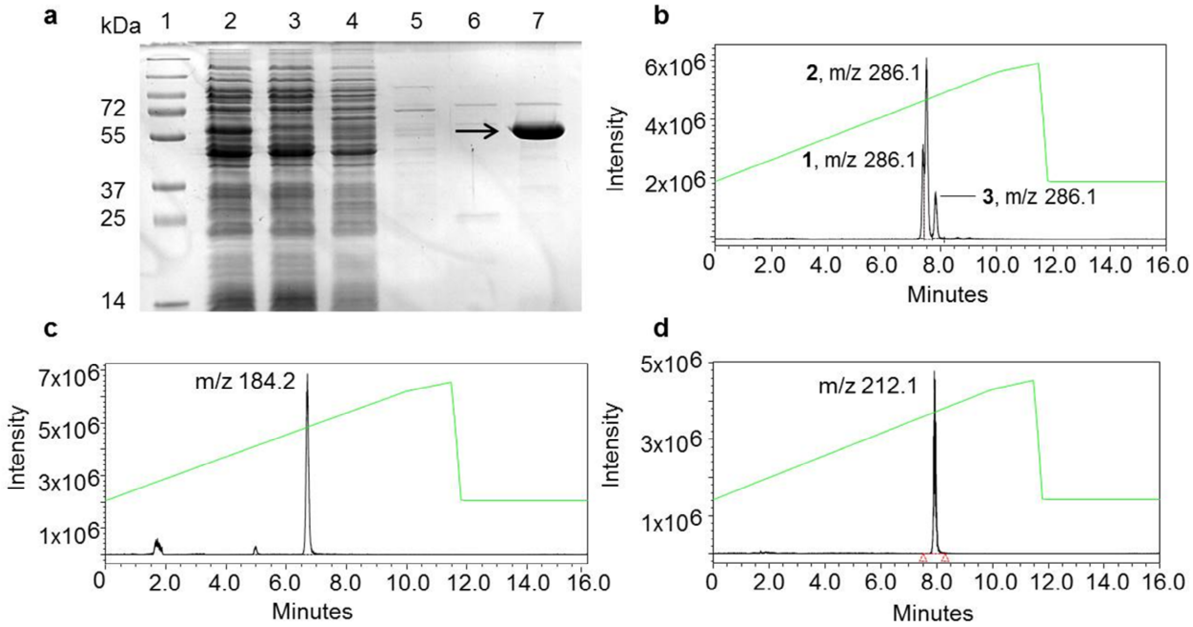

Supplementary Fig. S2: Purification and specificity of recombinant piperamide synthase. a The recombinant enzyme (marked by an arrow) was purified by affinity chromatography. **b** Product formation with piperoyl-CoA and piperidine; **c** Product formation with hexanoyl CoA and piperidine**; d** Product formation with octanoyl CoA and piperidine. Whereas in the case of piperoyl-CoA a mixture of three piperine isomers were detected by LC-UV/Vis and LC-ESI-MS detection (1, 2, and piperine marked as 3), a single product was produced in the case of hexanoyl-CoA (**c**), and octanoyl-CoA (**d**) identified as hexanoylpiperidine and octanoylpiperidine, respectively based on the corresponding mass signals at *m/z* 184.2 $[M+H]^+$ and *m/z* 212.1 $[M+H]^+$.

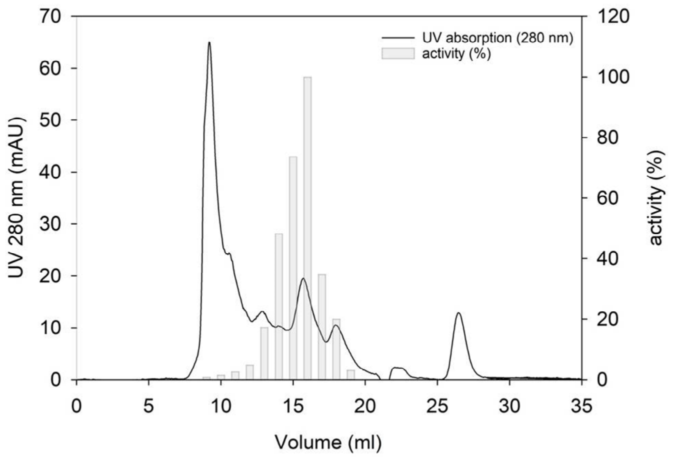

Supplementary Fig. S3: Size exclusion chromatography of a partly purified piperine synthase preparation. A Superdex 200 Increase 30/100 column (Cytiva, Freiburg, Germany) at a flow rate of 0.35 ml/min was used to correlate enzyme activity with molecular mass. Active, recombinant piperine synthase elutes after 16 ml as a monomer of ~ 50 kDa. Activity is marked by grey bars. No activity is observed with enzymes of higher, oligomeric states. Buffer includes 10 % glycerol, 150 mM NaCl, 1 mM DTT and 20 mM potassium phosphate at pH 7.5.

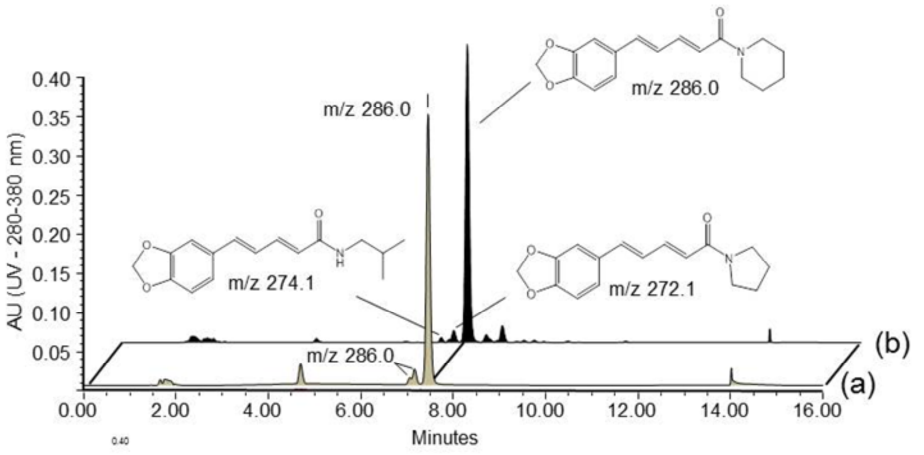

Supplementary Fig. S4: LC-MS analyzed methanolic extract of commercially available black pepper (*Kampot* variety) compared to piperine. a Piperine standard **b** Black peppercorns were ground to a fine powder, dissolved in 100 % MeOH at a concentration of 1 mg/ml, and were directly analyzed by LC-ESI-MS. The product profile recorded by UV/Vis shows the predominant presence of piperine at m/z 286 $[M+H]^+$ as in the case of a standard of commercially available piperine (a) and minor amounts of piperoylpiperidine (*m/z* 272.1) $[M+H]^+$ and piperoylisobutylamine (*m/z* 274.1) $[M+H]^+$.

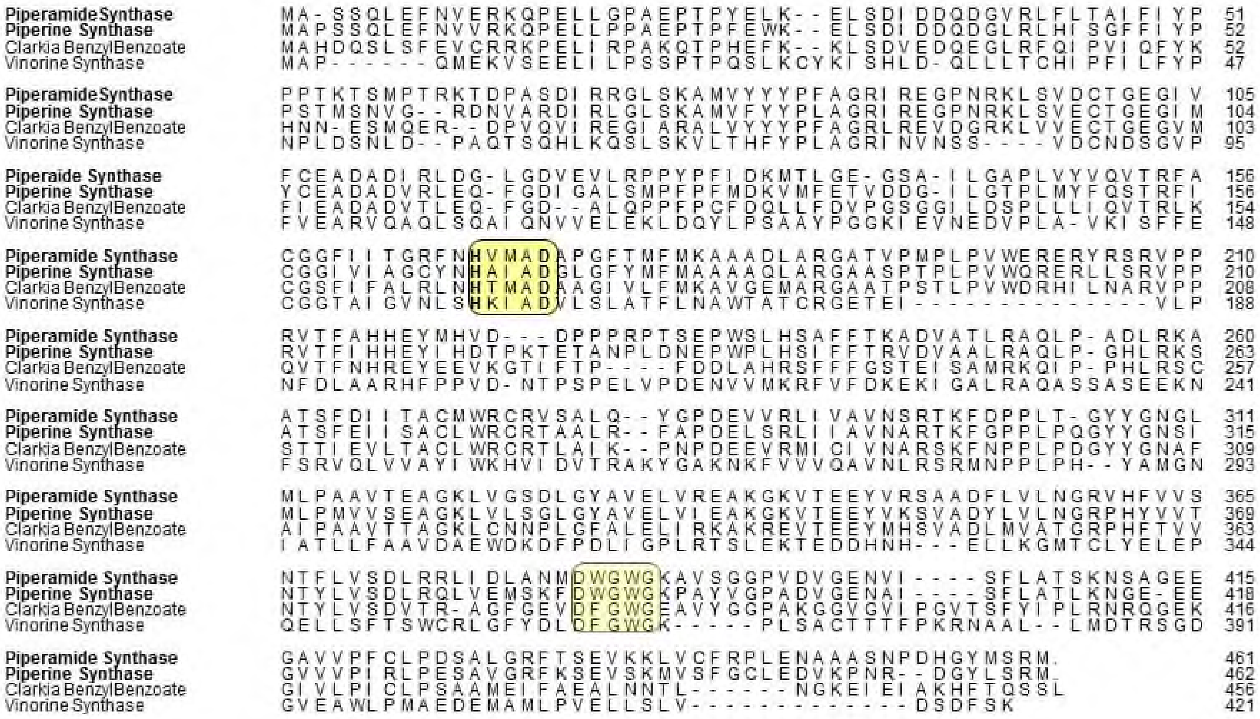

Supplementary Fig. S5: Sequence comparison of piperine and piperamide synthase with selected BAHD-type acyltransferases. Piperine synthase was compared to piperamide synthase and to the sequence with highest identity from *Clarkia brewerii*, benzylbenzoate acyltransferase[30] as well as vinorine synthase[32], the first crystallized BAHD-type acyltransferase. The catalytically relevant histidine and aspartate in the HXXXD box are conserved in all BAHD-protein sequences. The structurally relevant DFGWG motif, present in vinorine synthase and benzylbenzoate acyltransferase is slightly modified in both black pepper enzymes to DWGWG.

Supplementary Tables

Supplementary Table S1 Most abundant fruit stage I and stage II transcripts illustrate the rapid rise of specialized metabolism. The overall most abundant transcript in the fruit encoded a BURP-protein of unknown function. The terpene synthase 10-like transcript of specialized metabolism ranked number 5 of all transcripts identified at stage II of fruit development. This indicates the strong initiation of pathways of specialized metabolism at the onset of piperine accumulation. Transcripts encoding piperine synthase and piperamide synthase, both among the Top 5 candidates (green) are highlighted in bold. (*tpm = transcripts/million).

No.	Trinity ID	sprot_blastX - based annotation	Stage I tpm*	Stage II tpm*
Top	TRINITY_DN56430_c3_g3	BURP domain-containing protein 3	14781	17785
1	TRINITY_DN49139_c0_g3	Terpene synthase 10	199	1091
**2**	**TRINITY_DN43391_c4_g4**	**BAHD - Piperine Synthase**	**194**	**699**
3	TRINITY_DN53695_c0_g9	Cheilanthifoline synthase	157	549
4	TRINITY_DN60552_c4_g3	Chalcone synthase 3-like	648	476
**5**	**TRINITY_DN43391_c4_g1**	**BAHD - Piperamide Synthase**	**121**	**464**
6	TRINITY_DN53695_c0_g2	(S)-canadine synthase	127	444
7	TRINITY_DN58395_c1_g1	Acyl-coenzyme A oxidase 4	164	411
8	TRINITY_DN53669_c1_g4	Benzyl alcohol O-benzoyltransferase	116	351
9	TRINITY_DN43391_c4_g5	Benzyl alcohol O-benzoyltransferase	113	333
10	TRINITY_DN51211_c1_g2	Alpha-terpineol synthase, chloroplastic	108	328
11	TRINITY_DN42232_c1_g1	Adenylate isopentenyltransferase 3	336	328
12	TRINITY_DN53669_c1_g1	Benzyl alcohol O-benzoyltransferase	100	288
13	TRINITY_DN60552_c3_g1	Chalcone synthase 3	91	282
14	TRINITY_DN52087_c0_g2	Beta-cubebene synthase	98	270
15	TRINITY_DN52087_c0_g1	5-epi-aristolochene synthase 2	158	232
16	TRINITY_DN60552_c4_g2	Chalcone synthase 2	271	221
17	TRINITY_DN58865_c2_g2	Probable terpene synthase 3	210	210
18	TRINITY_DN44102_c0_g1	Flavanone 3-dioxygenase	190	190
19	TRINITY_DN59185_c3_g1	Probable terpene synthase 3	169	190
20	TRINITY_DN58916_c2_g1	Chalcone synthase	177	184
21	TRINITY_DN53695_c0_g5	Cheilanthifoline synthase	50	183
22	TRINITY_DN43338_c0_g1	Ornithine decarboxylase	128	160
23	TRINITY_DN60936_c1_g3	3-oxoacyl-[acyl-carrier-protein] synthase I	47	155
24	TRINITY_DN41616_c1_g1	Chalcone synthase 2	57	152
25	TRINITY_DN59185_c3_g3	5-epi-aristolochene synthase 2	44	134
26	TRINITY_DN59201_c1_g1	Dihydroflavonol 4-reductase	147	130
27	TRINITY_DN59329_c3_g1	Cytochrome P450 84A1 - FAH	102	105
28	TRINITY_DN44102_c0_g2	Naringenin,2-oxoglutarate 3-dioxygenase	109	100
29	TRINITY_DN48128_c4_g1	Anthocyanidin reductase	137	95
30	TRINITY_DN58865_c2_g3	Beta-cubebene synthase	50	67

Supplementary Table S2 Primers for cloning and qRT-PCR of piperine synthase and piperamide synthase.

Primer annotation	Sequence
Piperine synthase forward	5´- CATCATATGGCGCCTTCTTCTCAACTTG -3´
Piperine synthase reverse	5´- CATGGATCCTTACATGCGGGAAAGGTATCCATC -3´
Piperamide synthase forward	5´- CAT**CATATGG**CTTCTTCTCAGCTCGAATTC -3´
Piperamide synthase reverse	5´- CATGGATCCTTACATGCGGGACATGTACCCATG -3´
qP-BAHD1-for (Piperamide synthase)	5´- GAAAGCGGCAACCTCCTTTG -3´
qP-BAHD1-rev (Piperamide synthase)	5´- AACTTGGTCCGGGAGTTGAC-3´
qP-BAHD2-for (Piperine synthase)	5´- TTGGCGATATCGGAGCACTC-3´
qP-BAHD2-rev (Piperine synthase)	5´- CGATCCCGCCGCAAATAAAG -3´
qP-elF2B-for	5´- CAATGCTTGAAGGGCAGTCG -3`
qP-elF2B-rev	5`- AACACAGATGCAGCTCCCAA -3`
