# Supplementary material for: Identification and characterization of piperine synthase from black pepper, Piper nigrum L
Source: Commun Biol. 2021 Apr 8;4:445. doi: 10.1038/s42003-021-01967-9 (PMC8032705; doi:10.1038/s42003-021-01967-9)
Supplement: Supplementary file 3 — Description of Additional Supplementary Files [file 42003_2021_1967_MOESM3_ESM.pdf]

Description of Additional Supplementary Files

File name: Supplementary Data 1

Description: Sequence information of all genes aligned in Figure 6.
