# Supplementary material for: Identification and characterization of piperine synthase from black pepper, Piper nigrum L
Source: Commun Biol. 2021 Apr 8;4:445. doi: 10.1038/s42003-021-01967-9 (PMC8032705; doi:10.1038/s42003-021-01967-9)
Supplement: Supplementary file 5 — Reporting Summary [file 42003_2021_1967_MOESM5_ESM.pdf]

nature research

Corresponding author(s): Thomas Vogt

Last updated by author(s): Feb 24, 2021

Reporting Summary

Nature Research wishes to improve the reproducibility of the work that we publish. This form provides structure for consistency and transparency in reporting. For further information on Nature Research policies, see our Editorial Policies and the Editorial Policy Checklist.

Statistics

For all statistical analyses, confirm that the following items are present in the figure legend, table legend, main text, or Methods section.

n/a	Confirmed	
☐	☒	The exact sample size (*n*) for each experimental group/condition, given as a discrete number and unit of measurement
☐	☒	A statement on whether measurements were taken from distinct samples or whether the same sample was measured repeatedly
☐	☒	The statistical test(s) used AND whether they are one- or two-sided<br>*Only common tests should be described solely by name; describe more complex techniques in the Methods section.*
☒	☐	A description of all covariates tested
☒	☐	A description of any assumptions or corrections, such as tests of normality and adjustment for multiple comparisons
☐	☒	A full description of the statistical parameters including central tendency (e.g. means) or other basic estimates (e.g. regression coefficient) AND variation (e.g. standard deviation) or associated estimates of uncertainty (e.g. confidence intervals)
☒	☐	For null hypothesis testing, the test statistic (e.g. *F*, *t*, *r*) with confidence intervals, effect sizes, degrees of freedom and *P* value noted<br>*Give P values as exact values whenever suitable.*
☒	☐	For Bayesian analysis, information on the choice of priors and Markov chain Monte Carlo settings
☒	☐	For hierarchical and complex designs, identification of the appropriate level for tests and full reporting of outcomes
☒	☐	Estimates of effect sizes (e.g. Cohen's *d*, Pearson's *r*), indicating how they were calculated

Our web collection on statistics for biologists contains articles on many of the points above.

Software and code

Policy information about availability of computer code

Data collection	Sequencing (Illumina HiSeq2000)and demultiplexing of qPCR data was carried out by GATC BIOTECH (Konstanz).<br>Trinity was used for RNAseq-assembly (http://TrinityRNASeq.sourceforge.net)
Data analysis	Data mining and statistical analysis was performed in R (version 3.6.2).<br>Generation and analysis of HPLC data was performed with Waters Empower Software Version 3<br>RT-qPCR data were analyzed by CFX real time software system 3.1 (BioRad).<br>For data calculations Office Excel 2013 and Sigma Plot (Systat) 13.0/14.0 was used.

For manuscripts utilizing custom algorithms or software that are central to the research but not yet described in published literature, software must be made available to editors and reviewers. We strongly encourage code deposition in a community repository (e.g. GitHub). See the Nature Research guidelines for submitting code & software for further information.

Data

Policy information about availability of data

All manuscripts must include a data availability statement. This statement should provide the following information, where applicable:

- Accession codes, unique identifiers, or web links for publicly available datasets
- A list of figures that have associated raw data
- A description of any restrictions on data availability

All data are stored in a public repository: www.radar-service.eu as listed in the manuscript. Access will be provided by DOI: (DOI) 10.22000/400.

Field-specific reporting

Please select the one below that is the best fit for your research. If you are not sure, read the appropriate sections before making your selection.

☒ Life sciences ☐ Behavioural & social sciences ☐ Ecological, evolutionary & environmental sciences

For a reference copy of the document with all sections, see nature.com/documents/nr-reporting-summary-flat.pdf

Life sciences study design

All studies must disclose on these points even when the disclosure is negative.

Sample size	Sample size was chosen based on randomly selecting invidicual fruits from 10 different cutting grown at the same location, all cutting from a single specimen. For RNAseq Analysis triplicate measurements from all samples to be compared were made<br>For organ specific metabolite content, individual fruits at differential development were chosen. qRT-PCR analysis was performed in triplicates.
Data exclusions	No data were excluded
Replication	RNAseq-data sets were obtained once and not reproduced again. We compared different organs for sequence abundance to select for individual organ and developmental specific sequences. In principle, we used qRT-PCR to verify and also quantify the data obtained from RNA-seq.
Randomization	All samples in the RNA-seq analysis were picked randomly from 10 different plants. The only requirement for the fruits were that they were at the same developmental stage. This also holds true for the content of piperine. Developmental stages were selected based on days after anthesis which was recorded for each spadix separately.
Blinding	Blinding was not relevant to this study. Random selection based on the same developmental stage was performed.

Reporting for specific materials, systems and methods

We require information from authors about some types of materials, experimental systems and methods used in many studies. Here, indicate whether each material, system or method listed is relevant to your study. If you are not sure if a list item applies to your research, read the appropriate section before selecting a response.

Materials & experimental systems

n/a	Involved in the study
☒	☐ Antibodies
☒	☐ Eukaryotic cell lines
☒	☐ Palaeontology and archaeology
☒	☐ Animals and other organisms
☒	☐ Human research participants
☒	☐ Clinical data
☒	☐ Dual use research of concern

Methods

n/a	Involved in the study
☒	☐ ChIP-seq
☒	☐ Flow cytometry
☒	☐ MRI-based neuroimaging
